# Vasovagal Oscillations and Vasovagal Responses Produced by the Vestibulo-Sympathetic Reflex in the Rat

**DOI:** 10.3389/fneur.2014.00037

**Published:** 2014-04-04

**Authors:** Sergei B. Yakushin, Giorgio P. Martinelli, Theodore Raphan, Yongqing Xiang, Gay R. Holstein, Bernard Cohen

**Affiliations:** ^1^Department of Neurology, Icahn School of Medicine at Mount Sinai, New York, NY, USA; ^2^Department of Computer and Information Sciences, Brooklyn College of the City University of New York, Brooklyn, NY, USA; ^3^Department of Neuroscience, Icahn School of Medicine at Mount Sinai, New York, NY, USA

**Keywords:** syncope, wavelet analysis, sinusoidal galvanic vestibular stimulation, rat, isoflurane anesthesia, otolith

## Abstract

Sinusoidal galvanic vestibular stimulation (sGVS) induces oscillations in blood pressure (BP) and heart rate (HR), i.e., vasovagal oscillations, as well as transient decreases in BP and HR, i.e., vasovagal responses, in isoflurane-anesthetized rats. We determined the characteristics of the vasovagal oscillations, assessed their role in the generation of vasovagal responses, and determined whether they could be induced by monaural as well as by binaural sGVS and by oscillation in pitch. Wavelet analyses were used to determine the power distributions of the waveforms. Monaural and binaural sGVS and pitch generated vasovagal oscillations at the frequency and at twice the frequency of stimulation. Vasovagal oscillations and vasovagal responses were maximally induced at low stimulus frequencies (0.025–0.05 Hz). The oscillations were attenuated and the responses were rarely induced at higher stimulus frequencies. Vasovagal oscillations could occur without induction of vasovagal responses, but vasovagal responses were always associated with a vasovagal oscillation. We posit that the vasovagal oscillations originate in a low frequency band that, when appropriately activated by strong sympathetic stimulation, can generate vasovagal oscillations as a precursor for vasovagal responses and syncope. We further suggest that the activity responsible for the vasovagal oscillations arises in low frequency, otolith neurons with orientation vectors close to the vertical axis of the head. These neurons are likely to provide critical input to the vestibulo-sympathetic reflex to increase BP and HR upon changes in head position relative to gravity, and to contribute to the production of vasovagal oscillations and vasovagal responses and syncope when the baroreflex is inactivated.

## Introduction

The vasovagal response is usually defined as the development of inappropriate cardiac slowing and arteriolar dilatation, resulting in a sudden drop in blood pressure (BP). The bradycardia is thought to result from sudden augmentation of efferent vagal activity and the hypotension is attributed to a sudden reduction of sympathetic activity that relaxes the arterial resistance vessels (1). The vasovagal response can result in vasovagal syncope (a faint), which is due to a loss of appropriate blood flow to the brain. Normal cardiovascular function is restored and consciousness is regained following vasovagal syncope, if subjects are brought to a recumbent position. Fainting due to severe anxiety, pain, and blood loss has been known for centuries (2) and a faint culminates in drop of both BP and heart rate (HR) that slowly recover over minutes (1, 3). However, the endogenous physiological trigger(s) for the precipitous decline in BP and HR and the mechanics of the recovery of normal function are not clear (4–6). We have recently shown that vasovagal responses can be induced by vestibular activation using sGVS (7, 8). In this paper, we utilize a novel analytical analysis using wavelet decomposition of BP and HR waveforms to determine the range of frequencies and types of vestibular activation that could be important in initiating the vasovagal response.

Normally, BP is maintained at a stable level by feedback through the baroreflex pathway. One mechanism by which the baroreflex stabilizes BP in humans is by activating muscle sympathetic nerves in the legs to constrict peripheral arteries when BP falls (9, 10). Increases in muscle sympathetic nerve activity (MSNA) also occur upon arising, in order to prevent blood from pooling in the legs. This process is initiated through the vestibulo-sympathetic reflex (VSR) (6, 9, 11), which detects the change in head and body position and conveys this information to the sympathetic pathways controlling BP and HR. However, the processes underlying vestibular interactions with the baroreflex, especially those that initiate vasovagal syncope and subsequent recovery of function are unclear. MSNA is abruptly inhibited at the onset of a vasovagal response (12, 13). This suggests that there is a loss of baroreflex feedback, which could be a significant factor in initiating the vasovagal response and syncope. Consistent with this, baroreflex sensitivity is decreased in subjects that develop vasovagal syncope in response to tilt and lower body negative pressure (3, 14). How the baroreflex is altered, leading to the generation of vasovagal responses, is still not known.

Many frequencies are encompassed in BP and HR. In addition to the large changes in BP that can be attributed to systoles and diastoles, respiratory sinus arrhythmia reflects the expansion and contraction of the chest during breathing. There are also small, low frequency oscillations in BP, called Mayer waves, which are just below the respiratory rate (15) and reflect delays in the baroreflex feedback loop or resonance of the transfer function at a particular frequency [see Ref. (16) for review]. The Mayer wave frequency is 0.1 Hz in humans (17) and 0.4 Hz in rats (18).

When the baroreflex loop is disrupted, BP oscillates at lower frequencies (0.04–0.05 Hz) (19). Oscillations at similar low frequencies can also occur in response to blood loss (20). Similar low frequency oscillations in BP and HR have also been produced through the VSR in response to sinusoidal galvanic vestibular stimulation (sGVS) (7, 8). In the present report, these low frequency modulations in BP and HR are referred to as vasovagal oscillations (21). In some instances, sGVS induces a substantial fall in BP and HR, which recovers over several minutes. We define this fall as the “transient” component of the vasovagal response, which is the precursor for vasovagal syncope. It has been proposed that vasovagal syncope is dependent on the low frequency oscillations in BP (22). Two goals of the present study were to define the characteristics of vasovagal oscillations more precisely, and to determine their relationship to the generation of vasovagal responses.

Recent studies in isoflurane-anesthetized rats have shown that vasovagal responses can be generated by static tilts as well as by low frequency sGVS (8). Although anesthetized animals cannot faint, vasovagal responses in the rat can be a useful animal model of human vasovagal responses (7, 8). Presumably, the enhanced susceptibility to vasovagal responses in anesthetized rats is related to the reduced sensitivity of the baroreflex due to isoflurane anesthesia (23).

Vasovagal oscillations induced by sGVS in rats have prominent double oscillations, i.e., BP and HR are modulated at twice the stimulus frequency (7, 8). Similar double oscillations occur in MSNA induced by sGVS in humans (24, 25). These authors attribute the double oscillations to cathodal activation of the labyrinths, although these harmonics may arise in otolith units with specific polarizations. A third goal of the present study was to determine the likely origin of the double oscillations using monaural stimulation and oscillation in pitch. We also developed a novel analytical approach using a wavelet decomposition of the BP and HR waveforms induced by sGVS and oscillation in pitch to determine the contribution of the harmonics to the initiation of vasovagal response and syncope.

## Materials and Methods

Six adult, male Long-Evans rats (Harlan Laboratories, MA, USA), 400–500 g, were used in this study. All experiments were approved by the Institutional Animal Care and Use Committee at Mount Sinai. Vasovagal oscillations were synchronous low frequency modulations in BP and HR. Vasovagal responses were characterized by a “transient” component, i.e., rapid drops in BP and HR of 25 mmHg and 25 beats per minute (bpm), respectively, that slowly recovered over minutes. Vasovagal oscillations and vasovagal responses could be induced in each of the six rats.

Surgery and testing were conducted under isoflurane anesthesia (4% induction, 2% maintenance with oxygen). A telemetric BP sensor (DSI, MN) was implanted in the abdominal aorta during aseptic surgery. During animal preparation and experiments, the animals were kept on a heating pad at 37°C. The temperature was controlled by feedback from a rectal thermometer. Further details of the implantation and surgery have been provided previously (7, 8, 26).

Sinusoidal galvanic vestibular stimulation was generated by a computer-controlled stimulator. Current was delivered via subdermal needle electrodes placed in front of and behind each ear. The posterior electrodes were located over the mastoids and the anterior electrodes over the temporo-mandibular joints. Stimulation currents were 3 mA at frequencies between 0.025 and 0.5 Hz given continuously for 1–5 min with at least 15 min rest between stimuli.

To determine the frequencies of sGVS that were most likely to produce vasovagal responses, three rats were stimulated with sGVS at frequencies between 0.025 and 2 Hz in a pseudo-random sequence (0.025 Hz for 5 cycles, 2 Hz for 100 cycles, 0.5 Hz for 25 cycles, 0.1 Hz for 10 cycles, 1 Hz for 50 cycles, 0.05 Hz for 10 cycles, and 0.2 Hz for 10 cycles). If a vasovagal oscillation and/or a vasovagal response were induced, there was a 15 min interval until the next stimulus. If neither a vasovagal oscillation nor a vasovagal response were induced, at least 100 s elapsed between stimuli. The three rats were tested 27 times over 7 days. Rats were also stimulated with single sines of current that were given at 2 min intervals. In addition, the rats were oscillated ±50° and ±70° in pitch at frequencies between 0.025 and 0.2 Hz. It was not possible to oscillate the animals in pitch ±70° at 0.2 Hz, so they were pitched at ±50° at 0.2 Hz. At the end of the sinusoidal experiments, the rats were statically tilted 70° and held in this position until they developed a vasovagal response. If a vasovagal response developed or if there was no response after several minutes, they were then brought back to the prone position.

Intra-aortic BPs transduced by telemetric sensor were collected using a wand receiver (DSI, MN, USA). BP data, as well as the position of the tilt table and the current levels of sGVS were sampled at 1 kHz with 12 bit resolution (Data Translation, Inc., MA, USA). BP was continuously monitored and recorded. Using software developed in our laboratory, HR was computed offline from the systolic peaks of the BP signal, and was converted to an analog signal in bpm. There was no substantial difference in the variation of BP derived from systoles, diastoles, or mean BP. Therefore, systolic BPs were used to derive the measure of BP in this report. Breath rate was estimated for each experimental day for each rat from their respiratory sinus arrhythmia. For this, 50 time intervals were collected at the beginning and end of each experiment that included the inspiratory increases in BP. The breathing rate for six animals was 0.86 ± 0.09 Hz and varied from 0.38 to 1.41 Hz.

### Wavelet analysis

Blood pressure is a complex waveform comprised of systolic and diastolic phases whose amplitudes and frequency vary with time. HR is also a time varying signal composed of multiple frequencies. The sGVS and tilt stimuli are sinusoidal functions close to a single frequency over a limited time period. Both HR and BP oscillated in a sinusoidal fashion in response to sGVS, but neither were true sinusoids; instead, the responses were composed of many frequencies. Consequently, the time–frequency characteristics of the BP and HR functions were studied with a discrete wavelet analysis, which identified the contribution of particular bands of frequencies as a function of the time domain. It also optimized the time–frequency resolution in analyzing the BP and HR functions.

To determine how the frequency distribution of the response waveforms was spectrally distributed, it was necessary to ensure that the stimulus was confined to a single band of frequencies in the wavelet decomposition. This was done by resampling the stimulus signal so that its frequency was in the center of a band whose upper frequency limit was √ 2*stim_freq and the lower frequency limit was stim_freq/√ 2. Four low frequency bands were analyzed: activity in Band 12 and up, which covers an approximation band with a frequency of 0–0.018 Hz, indicated a transient response. The other three bands were: band 11 (0.018–0.035 Hz), Band 10 (0.035–0.071 Hz), and Band 9 (0.071–0.141 Hz). Activity in Bands 8 (0.141–0.282 Hz) and 7 (0.282–0.564 Hz) was minimal. Depending on the stimulus frequency used in an experiment, one of the bands (11) incorporated the stimulus frequency, while adjacent bands (10 and 9) incorporated the second and fourth harmonics of stimulation, i.e., they were centered at twice and four times the stimulus frequency.

The distribution of the power in each frequency band comprising the signal was used as a metric for determining how the stimulus generated activity at other frequencies. It was used as a basis for comparing the response to different stimuli. The power of each frequency band was computed as the average energy of the signal when it was reconstructed from frequency components in the band, calculated by (signal^2^/time). This was tested with three sinusoids of frequencies 0.025, 0.05, and 0.1 Hz. Each signal lasted 200 s and the original sampling interval was 16 ms to remove frequencies above 36.2 Hz, which were outside the range of interest. Using resampling and the Db12 wavelet analysis software, the leakage from the band associated with the stimulus frequency to other bands was less than 5% and a sinusoid at a single frequency had all of its energy in a single band of frequencies. This made it possible to determine how the power of the BP and HR responses was distributed to other bands by central processing.

The analysis was performed using Matlab (Mathworks, Inc., MA, USA). Standard deviations of wavelet-filtered responses for each frequency band were computed to compare results of wavelet decomposition of different data sets (27). The dominant peaks at the frequency of stimulation and twice the frequency of stimulation for the monaural and binaural stimuli were compared statistically using a Student’s paired *t*-test or a one way ANOVA with repeated measures applying a *post hoc* Bonferroni adjustment. Changes in BP and HR were significant at *p* < 0.05. Data are presented as mean ± 1SD throughout the manuscript.

## Results

**Figure 1:**
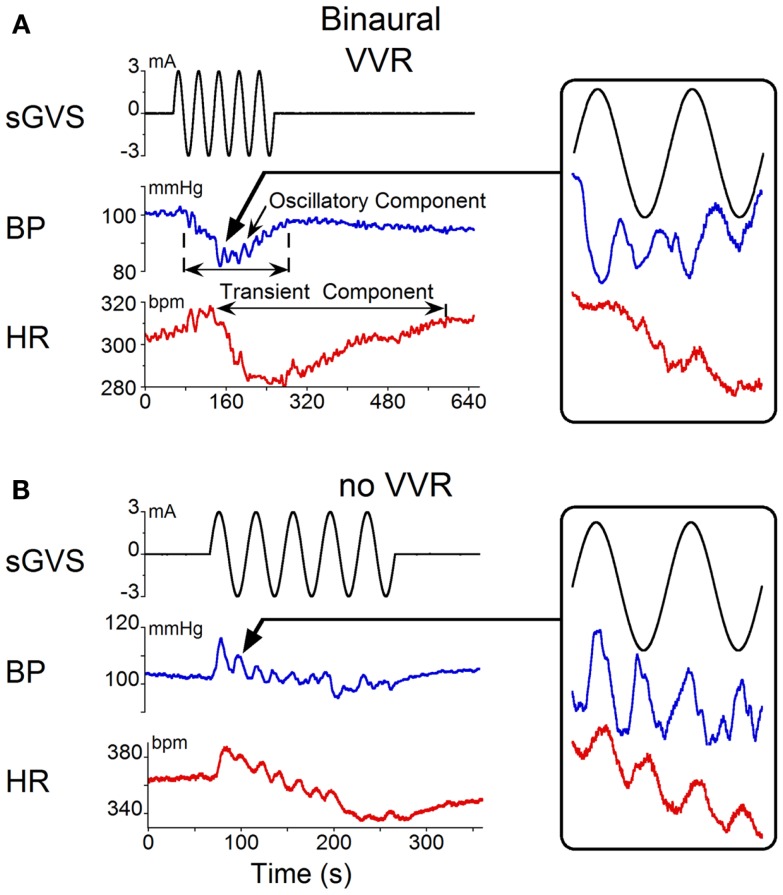
**Changes in blood pressure (BP) and heart rate (HR) induced by binaural 3 mA sGVS at 0.025 Hz**. **(A)** Vasovagal oscillations and a vasovagal response (VVR) induced by sGVS. Top trace is stimulus (black), middle trace is BP (blue), bottom trace is HR (red). The oscillatory component was at twice the stimulus frequency and was present before and during the transient response. The transient component was characterized by a steep drop in BP and HR that persisted for several minutes. The changes in HR were more pronounced than the changes in BP. Inset on the right is an expanded trace of two cycles (80 s) of sGVS. Arrow indicates location of the data expanded in the inset. BP and HR in the inset are not scaled, to illustrate oscillations at twice the stimulus frequency. **(B)** In another experiment, the same stimulus induced oscillations at twice the stimulus frequency in both HR and BP (see inset, showing two cycles of sGVS on an expanded trace). This stimulus induced a slight drop in HR, but no prolonged drop in BP.

Binaural sGVS at 0.025 Hz induced vasovagal oscillations both at the stimulus frequency and at twice the stimulus frequency, and transient drops in BP and HR in all six rats. An example of vasovagal oscillations at twice the stimulus frequency is shown in Figure 1A. There were concomitant transient drops in BP (blue) and HR (red) at the start of the transient component. This indicated that the animal was having a vasovagal response. For each 40 s cycle of binaural sGVS, there were two vasovagal oscillations (Figure 1A, inset). These double oscillations occurred in the periods preceding and during the transient drops in BP and HR, suggesting that they were likely to represent the input to the cardiovascular system from the VSR. In most instances, both HR and BP declined at some point during the stimulation, demonstrating the classical properties of a vasovagal response (1). BP and HR could be dissociated, however, illustrating the development of the vasovagal response. In one example, the initial decrease in BP was partially compensated by a rise in HR (Figure 1A). This is likely to represent an attempt of the baroreflex to compensate for the drop in BP, and is similar to episodes of pre-syncope noted by Julu et al. in humans (3). Such oppositely directed changes in BP and HR at the onset of vasovagal responses were typical for this animal, occurring in 80% of the induced vasovagal responses and were present in other animals as well. Following the drop in BP and the small rise in HR, both BP and HR then declined, while continuing to have a strong second harmonic component relative to the sGVS frequency. The amplitude of the changes in BP and HR were approximately 7 mm Hg and 7 beats per second, respectively.

Double harmonics during binaural stimulation at 0.025 Hz did not necessarily lead to the generation of a vasovagal response. Thus, in some experiments, stimulation of the same animal with sGVS at the same frequency as shown in Figure 1A did not generate a vasovagal response (Figure 1B), but there were still two oscillations of BP and HR for every cycle of sGVS (Figure 1B, inset). Induction of double oscillations without production of a vasovagal response was present in other animals as well.

The frequency of stimulation that was most likely to induce a vasovagal response was determined in three rats, which were tested with pseudo-random sets of sGVS from 0.025 to 2 Hz (see Materials and Methods for details). In the three animals, stimulation at 0.025 Hz induced vasovagal responses in 67% of stimulus presentations (18/27), and stimulation at 0.05 Hz induced vasovagal responses six times (6/27, 22%). Vasovagal responses were rarely induced at higher frequencies; once at 0.1 Hz (1/27, 4%) and once at 0.5 Hz (1/27, 4%). Thus, stimulation at 0.025 Hz had the highest probability of inducing vasovagal responses in these three rats. We also determined the mean susceptibility to develop vasovagal responses at 0.025 Hz in all six rats. The average susceptibility (number of sGVS stimulus presentations leading to a vasovagal response/total number of sGVS stimulus presentations) was 34 ± 21% (varying from 10 to 67%). Thus, although susceptibility varied among animals, all animals responded to stimulation at 0.025 Hz with generation of a vasovagal response at some time.

**Figure 2:**
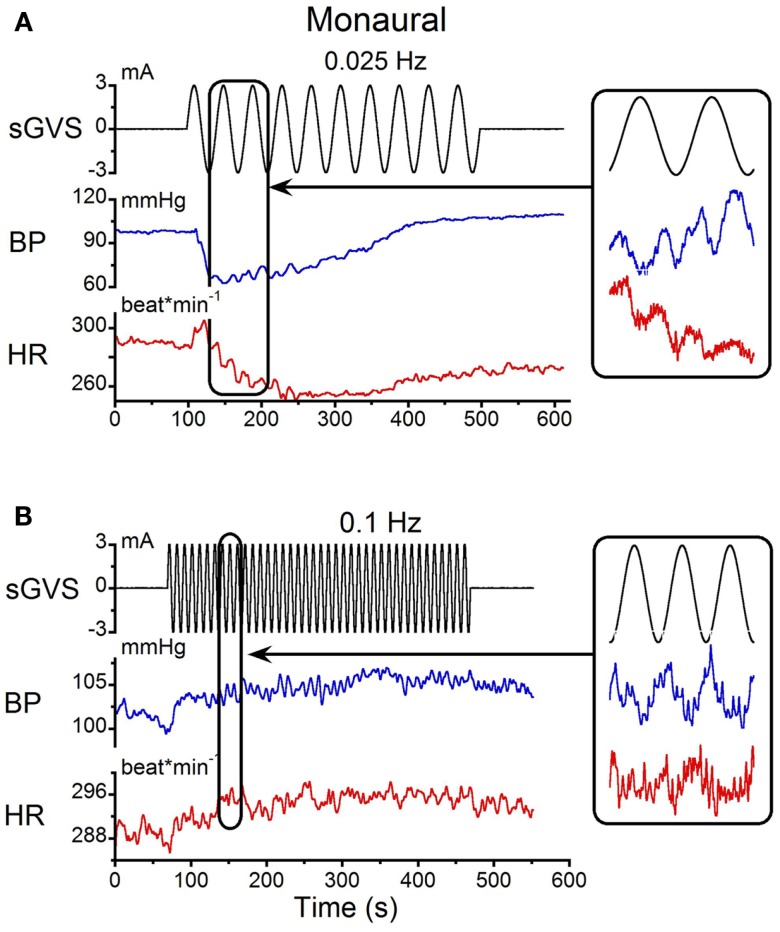
**Monaural sGVS induced by 3 mA sinusoid at 0.025 Hz (A) and 0.1 Hz (B)**. **(A)** The 0.025 Hz stimulus (black) induced transient decreases in systolic BP (blue) and HR (red). There were two oscillations in BP and HR for each stimulus cycle (expanded trace on right). **(B)** A higher stimulus frequency (0.1 Hz) induced only oscillatory components in systolic BP and HR at the frequency of stimulation (expanded trace on right).

The responses to monaural sGVS were compared with those from binaural stimulation. Monaural sGVS at 0.025 Hz produced the same frequencies of vasovagal oscillation as the binaural stimulus (Figure 2A; cf Figure 1A). The monaural stimulus also produced a characteristic vasovagal response that was the same as the vasovagal response induced by the binaural stimulus (Figure 2A). When monaural sGVS was given at higher frequencies, the amplitude of the induced oscillations was reduced, and vasovagal responses occurred only infrequently. Additionally, the second harmonics were not present at the higher frequencies, and only single oscillations in BP were induced by stimulation at 0.1 Hz and above (Figure 2B, inset). Therefore, the generation of the double harmonic (double oscillations) was dependent on the frequency of stimulation, and not on whether the stimulus was monaural or binaural.

**Figure 3:**
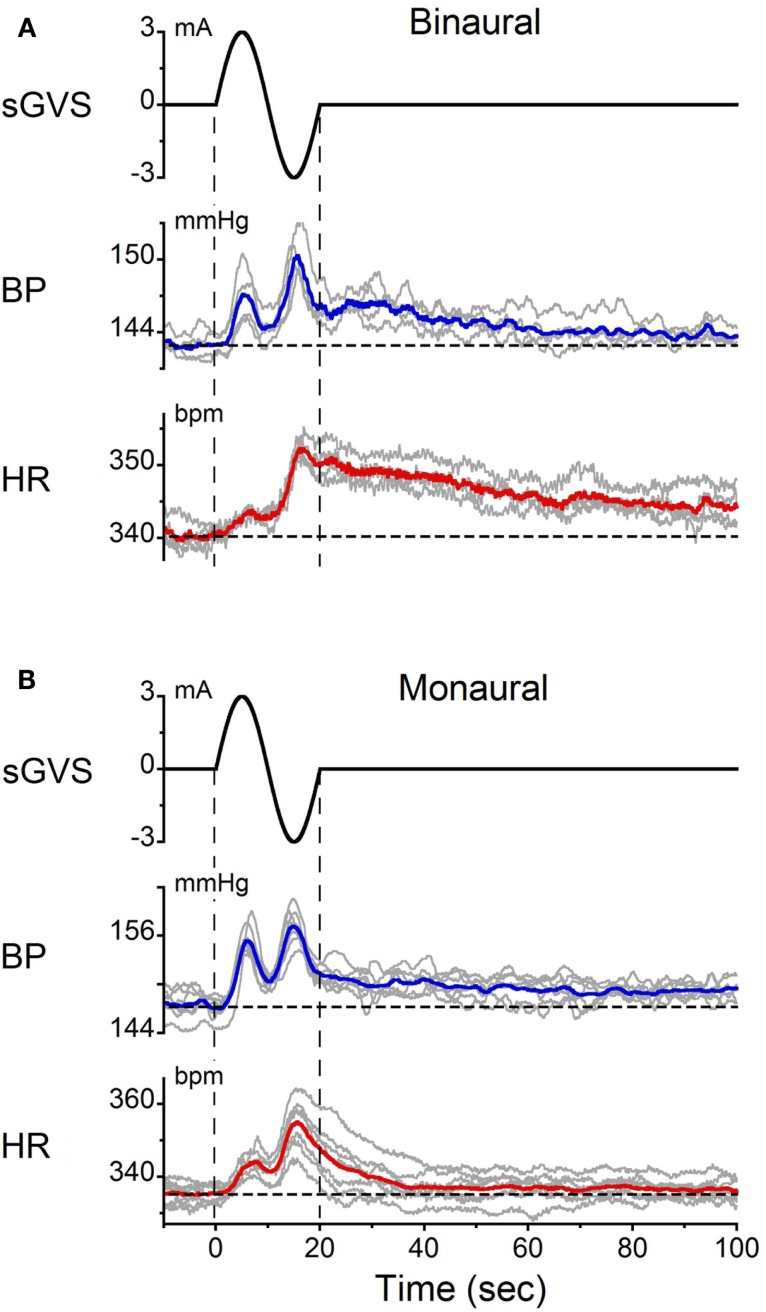
**Changes in BP and HR induced by single cycles of sGVS at 0.05 Hz (20 s) given (A) binaurally and (B) monaurally**. The stimulus was repeated 10 times at 2 min intervals. The data were synchronized from the onset of sGVS. Gray traces – responses to individual stimuli. Blue and red traces – averaged responses for BP and HR, respectively. Vertical dashed lines indicate the onset and offset of sGVS. Horizontal dashed lines show the average BP and HR at the onset of sGVS. Note the double oscillations induced by each sinusoid and the prolonged decline in BP [blue traces in **(A,B)**] and HR [red traces in **(A,B)**] over 120 s.

Single sinusoids of sGVS were given binaurally at 2 min intervals to determine whether they produced the same changes in BP and HR as trains of sGVS. It was also of interest to determine the average amplitudes and latencies of BP and HR induced by these stimuli. The responses to single sinusoids in one experiment are shown in Figure 3. The changes in BP and HR induced by multiple presentations of the stimulus were overlaid and averaged (Figure 3A). On average, BP rose from 143 to 147 mmHg in the first half of the sinusoid and from 145 to 150 mmHg during the second half. Although there was a tendency for the second BP peak to be slightly larger than the first, overall there was no statistically significant difference between the two phases of the response. BP then gradually declined to pre-stimulus levels over the next 120 s. The latency of the BP responses could not be determined accurately because of the slow rise and fall in current during each half cycle of stimulation. That is, the current rose or fell from 0 to ±3 mA over a 5 s period in each half of the sinusoid. Nevertheless, the time of the first significant (±3 SD) change in BP relative to the onset of stimulation could be determined. This varied from 1.1 to 2.0 s for both binaural and monaural stimulation; the average in the three tested animals was 1.5 ± 0.3 s.

In response to the same stimulus, HR increased from 340 to 345 bpm during the first half of the sinusoid and from 344 to 353 bpm in the second half. Changes in HR were somewhat larger during the second than during the first half of the sinusoid. HR then slowly declined back to baseline over 120 s. As with BP, the latency of the earliest changes in HR (±3 SD) could not be determined accurately. However, the latency of the peak changes in BP and HR were the same in all three rats (*p* = 0.131).

Double oscillations were also observed during monaural stimulation (Figure 3B). The average changes in BP and HR were 6.7 ± 1.8 mmHg and 6.2 ± 1.3 bpm, respectively. There was variation in the amplitudes of the first and second oscillations between trials, but when the responses were averaged over trials, the magnitude of the first and second peaks of the BP and HR responses for binaural and monaural stimulation were not significantly different (*p* > 0.05, ANOVA). These data demonstrate that similar BP and HR responses were induced by monaural and binaural stimulation, and that double oscillation could be produced by single sinusoids of sGVS.

**Figure 4:**
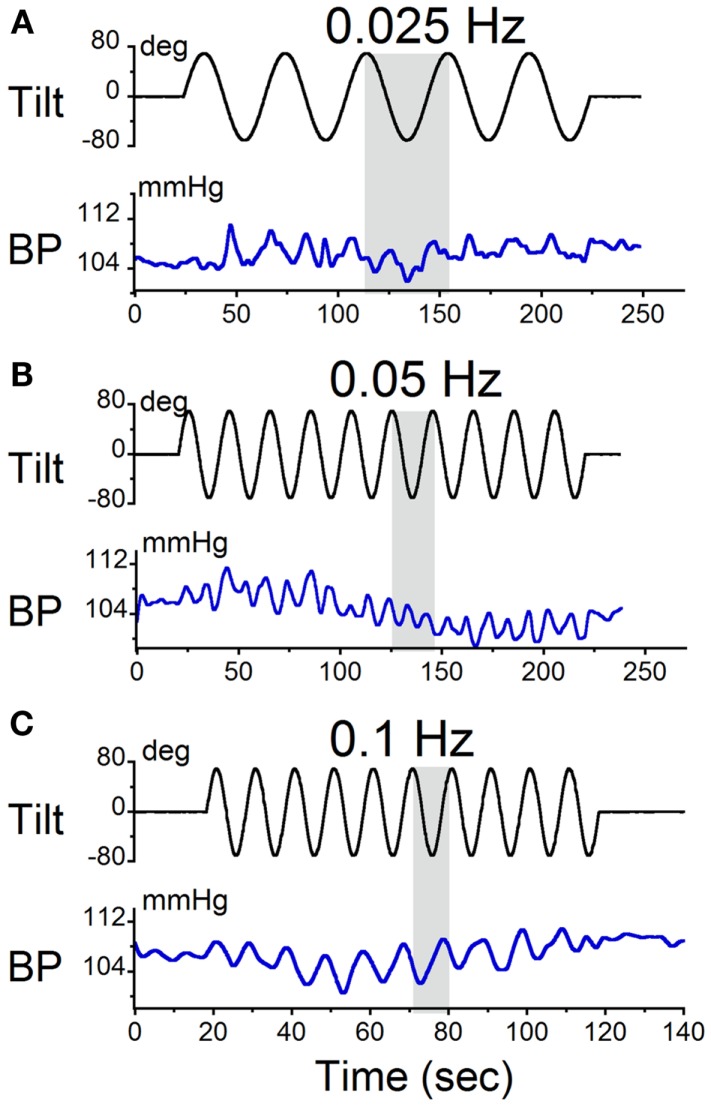
**Sinusoidal oscillations in pitch ±70° at (A) 0.025 Hz, (B) 0.05 Hz, and (C) 0.1 Hz**. The black trace in each panel is the tilt stimulus; the blue trace is systolic BP. The gray area indicates one tilt cycle. BP oscillated at twice the stimulus frequency at 0.025 and 0.05 Hz, but not at 0.1 Hz.

The animals were sinusoidally oscillated in pitch about a spatial horizontal axis at frequencies from 0.025 to 0.1 Hz. These frequencies were similar to those used during sGVS. The amplitudes of oscillation were ±70°(±0.9 g). The modulations in BP were at twice the stimulus frequency (Figures 4A,B). Consistent with the findings from sGVS (Figure 2B), stimulation at 0.1 Hz only produced oscillations at the stimulus frequency (Figure 4C). Vasovagal responses were rarely induced by oscillation at higher frequencies. Similar data were obtained in all six rats.

**Figure 5:**
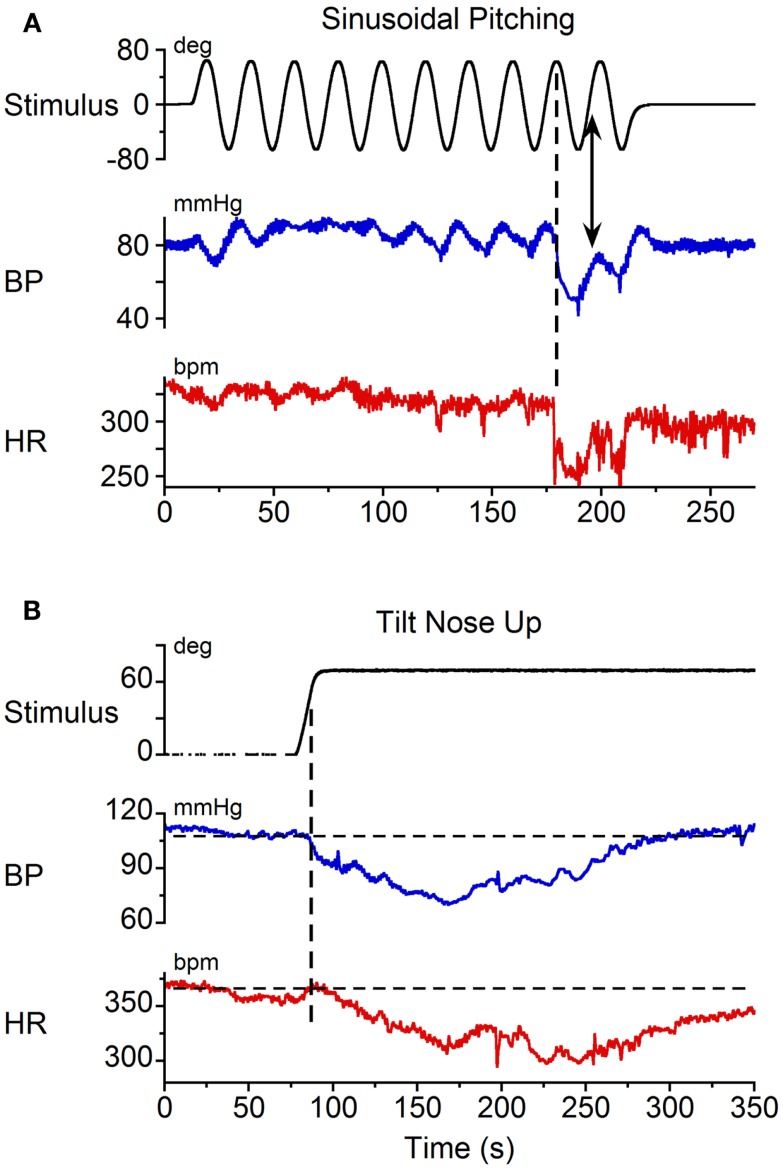
**Vasovagal responses induced by (A) sinusoidal pitching ±70° at 0.025 Hz and (B) static 70° nose-up body tilts**. Black trace: stimulus; blue trace: BP; red trace: HR. **(A)** A vasovagal response was induced by sinusoidal pitching. The onset of the transient changes in BP and HR occurred when the animal was approximately 70° nose-up, as indicated by the vertical dashed line. The vasovagal response, which was initiated when the animal was nose-up, was terminated as the animal approached the prone position. **(B)** A vasovagal response induced by static nose-up tilt of 70°.

Static nose-up tilts of 70°, which induce a change in linear acceleration of 0.9 g along the X–Z plane of the head and body, are an adequate stimulus to induce vasovagal responses (8). These responses can be terminated rapidly by bringing the animals to the prone position [cf. Figure 4 (8)]. Since some time is required after reaching the peak to generate the vasovagal response, we postulated that the animals did not remain in the upright position long enough to initiate a vasovagal response. Three lines of evidence support this hypothesis. First, as noted above, vasovagal responses can be terminated by rapidly bringing the animals from a tilted to a prone position. Second, data in Figure 5A demonstrate sinusoidal oscillation in a vasovagal responsive rat. A drop in BP was initially induced (Figure 5A, vertical dashed line), but was rapidly terminated as the rat was oscillated into the prone position (Figure 5A, arrow). Third, vasovagal responses could be induced in each of these animals by static 70° nose-up tilt (Figure 5B). These vasovagal responses were associated with production of large decreases in BP (40 mm Hg) and HR (65 bpm), i.e., in large vasovagal oscillations. From this, we conclude that if insipient vasovagal responses were induced, as in Figure 5A, they were rapidly terminated by the characteristics of the sinusoidal pitch.

### Wavelet analysis

**Figure 6:**
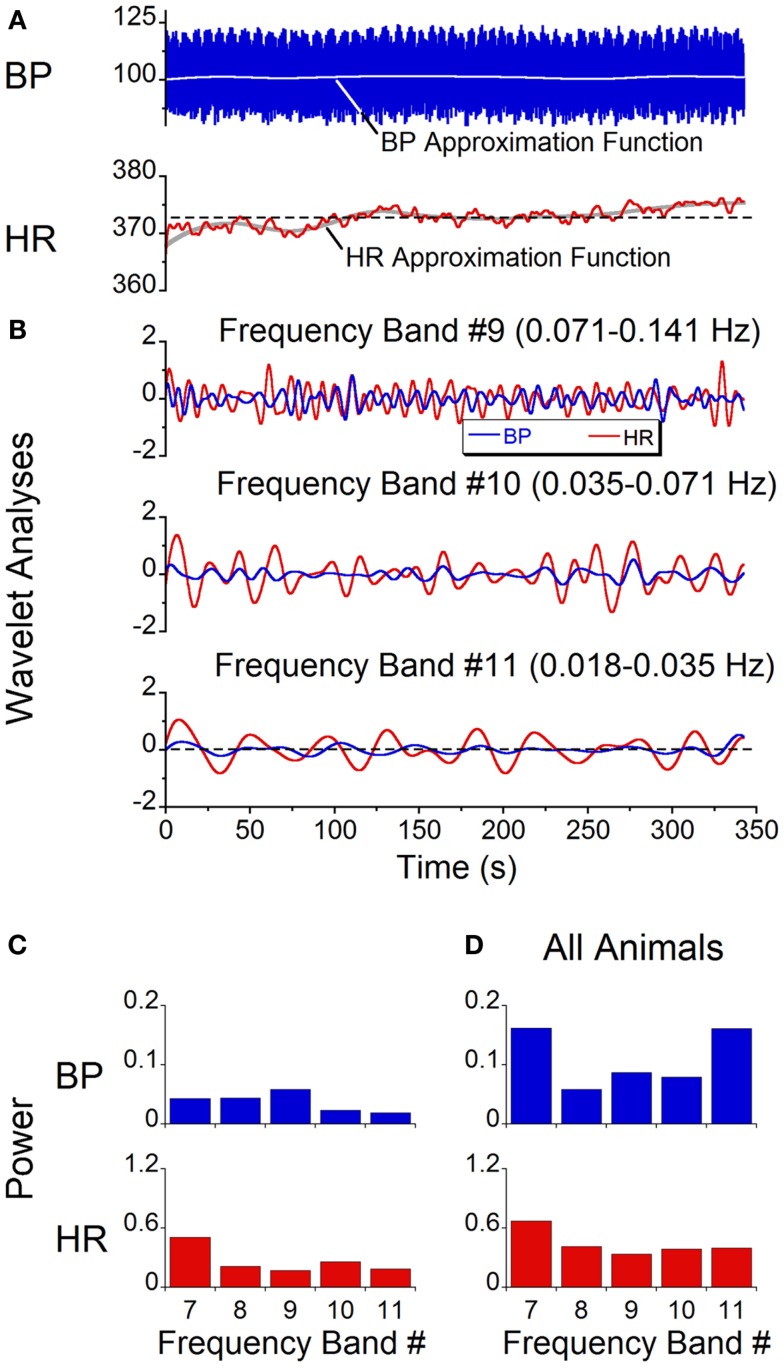
**Spontaneous oscillations in BP and HR with no vestibular stimulus**. **(A)** BP and HR recordings. The gray lines represent approximation function median values of BP (blue trace) and HR (red trace). **(B)** Wavelet decomposition of signals shown in **(A)** into individual frequency bands. BP (blue traces) is in millimeter of mercury and HR (red traces) is in beats per minute. **(C)** Power of individual bands of BP (blue) and HR (red) obtained from data shown in **(A)**. **(D)** Average power of BP (blue) and HR (red) across frequency bands 7–11 in all six rats.

While qualitative observations of the BP and HR waveforms brought into evidence the dominant and second harmonic components of the signals, BP and HR are comprised of multiple waveforms of different frequencies, and these waveforms vary over time. Wavelet analyses were performed to determine the time functions associated with specific bands of frequencies and the power distribution across bands. Initially, a wavelet decomposition of the responses with the animals at rest was done to establish a baseline. At rest, there were only low amplitude modulations of systolic BP and HR at 0.05 Hz (28) (Figure 6A). The wavelet decomposition reflected this low level of modulation across all of the low frequency bands (Figure 6B, Bands 9, 10, 11) and the power distribution was fairly uniform across all bands with a peak power on the order of 0.05 for BP and 0.5 for HR (Figure 6C). When all animals were included, while there was considerably more variability across animals, the average power across all bands was still low (≈0.15 for BP and ≈0.5 for HR, Figure 6D). There was no significant difference between the power levels across bands (ANOVA, *p* > 0.05). Thus, when there was no vestibular stimulation, the oscillations in BP and HR were of low amplitude with only small power in the bands outside those that included the heartbeats. We concluded that the amplitude of oscillation at any frequency was not significant unless it exceeded 0.5 of the total power.

**Figure 7:**
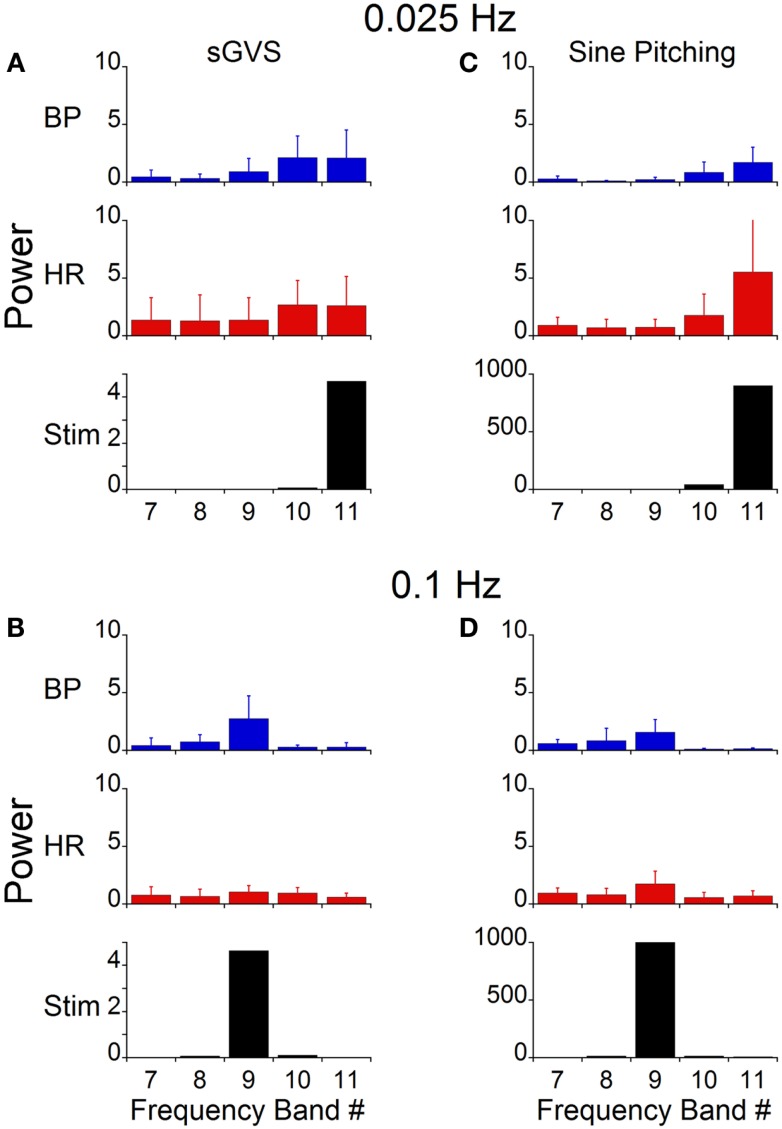
**Power of BP, HR, and stimulus of vasovagal oscillation in different frequency bands induced by sGVS and sinusoidal pitching ±70°**. The data were obtained at 0.025 Hz **(A,C)** and 0.1 Hz **(B,D)**. The stimulus power was in a single band [band 11 in **(A,C)**; band 9 in **(B,D)**]. Most of the power in BP and HR was at the stimulus and at twice the stimulus frequencies. The error bars represent one SD.

A wavelet decomposition of BP and HR was done when stimulating at 0.025 Hz in the six rats. When a vasovagal response was not induced, the maximal activation of the oscillations of BP and HR were limited to Bands 10 and 11, which contained the stimulus frequency and twice the stimulus frequency (Figure 7A). The activity in Bands 7–9 was negligible and was comparable to the baseline activity recorded without stimulation (Figure 6D). Although the induced waveforms had double oscillations, when the oscillations were decomposed, the component frequencies occurred at both the frequency of stimulation and at twice the frequency of stimulation. In contrast, the maximal power of the waveform induced at 0.1 Hz was at Band 9 (Figure 7B), i.e., at the frequency of stimulation, and the amplitude of the oscillations was considerably reduced. Moreover, there was no second harmonic. The response at 0.1 Hz was limited to Band 9, i.e., the band that contained the stimulus frequency. The modulation of HR was negligible in all bands at this stimulus frequency.

**Figure 8:**
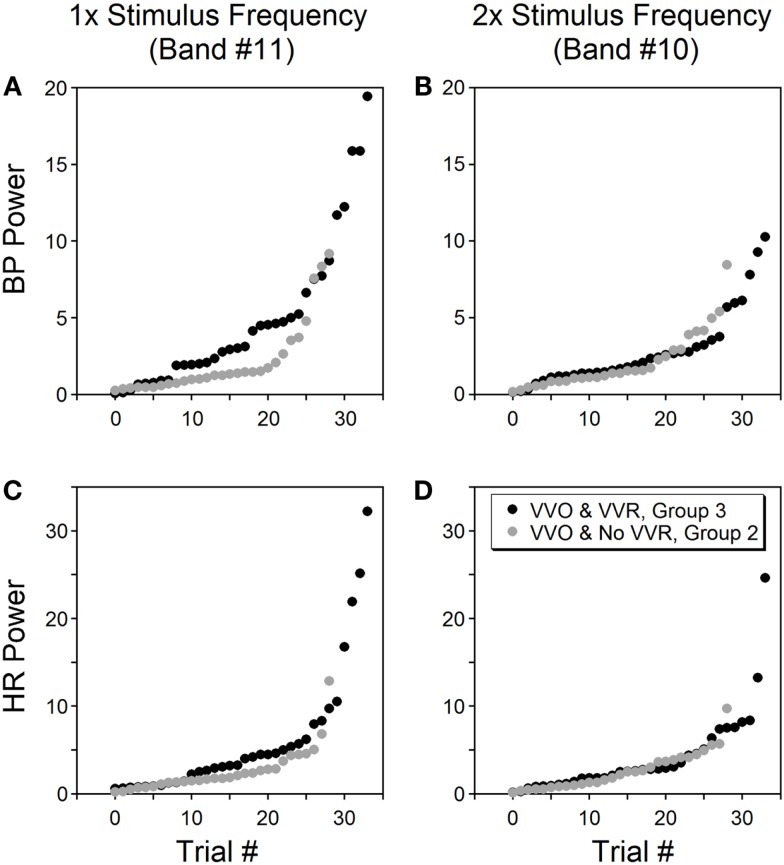
**Power of BP (A,B) and HR (C,D) at bands containing the stimulus frequency (left column) and twice the stimulus frequency (right column)**. Data were from six rats tested by sGVS at 0.025 Hz. Only trials that induced vasovagal oscillations (VVO) are shown, from which 34 trials (black circles, group 3) also induced vasovagal responses (VVR). The other 29 trials (gray circles, group 2) had vasovagal oscillations but no vasovagal responses. The abscissa (trial #) represents the order of each trial, is sorted on the magnitude of its power within a specific frequency band. The power in the stimulus frequency band was generally greater when there were vasovagal responses, more so for BP than for HR. However, the power was essentially the same in the double stimulus frequency band regardless of whether there were vasovagal responses or not.

The power in the bands containing once and twice the stimulus frequency were compared to determine whether the power distribution at low frequencies was correlated with the generation of vasovagal responses. The database consisted of 91 trials from six rats. The first group (composed of 28 trials) had no oscillatory components in BP and HR and no vasovagal response to sGVS at 0.025 Hz. The second group (29 trials) had substantial oscillatory components in BP and HR in response to sGVS, but vasovagal responses were not induced. The third group (34 trials) had substantial oscillations in BP and HR, i.e., vasovagal oscillations, and they developed concurrent vasovagal responses. The six rats were not equally distributed among the three groups. One rat was extremely susceptible to generation of a vasovagal response and fell into the third group. One rat was only occasionally susceptible and most of its responses fell into the first group. Four other rats were distributed through groups two and three, sometimes having just oscillations and other times developing a vasovagal response. The oscillations were induced at the stimulus frequency and at twice the stimulus frequency (Figure 8, Group 2, grey circles; Group 3, black circles). There was no difference in the second harmonic between Groups 2 and 3 in BP (*p* = 0.726, ANOVA with Bonferroni adjustment; Figure 8B) and HR (*p* = 0.18; Figure 8D). In contrast, there was a striking difference in the power of the first harmonic between the two groups in BP (*p* = 0.0038; Figure 8A) and HR (*p* = 0.0153; Figure 8C). Thus, there was a substantial increase in the power of the first harmonic when a vasovagal response was induced, regardless of individual susceptibility.

This experiment demonstrates that vasovagal oscillations could occur without induction of a vasovagal response, but that every vasovagal response was accompanied by a vasovagal oscillation. Although the power in the band containing the stimulus frequency and twice the frequency varied from experiment to experiment in Groups 2 and 3, the power of BP and HR at these frequencies was significantly larger than for Group 1, where no oscillations and no vasovagal responses occurred (*p* < 0.001).

The power at all frequency bands (1–11) was compared for Groups 1–3: Group 1, no vasovagal responses and no vasovagal oscillation; Group 2: only vasovagal oscillations; Group 3, vasovagal oscillation and vasovagal responses. When there was no stimulus, the maximal energy in Bands 8–11 was <0.5% (Figure 6). From this, we conclude that any response with a power below 0.5% is noise. The maximal power of ≈85% was in Bands 2–3 (4.5–18.1 Hz; Table 1). Activity in these bands reflects the expression of HR in BP. There was a substantial drop in activity in Band 1, which is above the range of BP variation from heartbeat (systole to diastole; 18.1–36.2 Hz; Table 1). Activity in Bands 8–11 (0.018–0.283 Hz) was largely dependent on whether a vasovagal response or vasovagal oscillations were induced (Table 1). When a vasovagal response was generated, the total activity in these bands was 9.3% of the total power. When only vasovagal oscillations were induced, the total activity was 4.8%, and when no vasovagal responses or oscillations were induced, activity in Bands 8–11 did not exceed the noise level. Thus, as the state of the rat changed from rest to vasovagal response, the activity in the low frequency bands increased from 0 to 4.8% of the total power, and then to 9.3%. Associated with this, there was a concomitant reduction in the power of the heartbeat and the percentage of the power in the band that reflected HR (2–3) was accordingly reduced from 90 to 87% and then to 80%. Consistent with the data shown in Figure 8, there was more power in the band containing the stimulus frequency than in the band containing twice the stimulus frequency, when vasovagal responses were induced. Activity in bands 3–7, which includes the breath rate (Band 6), was relatively stable throughout.

**Table 1:** **Power distribution induced by sGVS in three conditions: VVO and VVRs, only VVO, no VVO and no VVRs**.

Band no.	Frequency range (Hz)	VVR (*n* = 34)	No VVR	
			VVO (*n* = 29)	No VVO (*n* = 28)
1	18.102–36.204	2.19	2.24	2.69
2	9.051–18.102	17.65	20.77	22.41
3	4.525–9.051	61.74	65.72	68.14
4	2.263–4.525	7.68	3.82	4.02
5	1.131–2.263	0.40	0.91	0.26
6	0.566–1.131	0.92	1.43	2.06
7	0.283–0.566	0.11	0.33	0.08
8	0.141–0.283	0.12	0.24	0.05
9	0.071–0.141	0.52	0.70	0.10
10	0.035–0.071	2.90	1.99	0.10
11	0.018–0.035	5.78	1.85	0.09

*Data were obtained with sGVS at 0.025 Hz. Low frequency bands considered as vasovagal oscillation bands are marked in gray*.

The stimulus frequency during pitch was similar to that during sGVS, and was dominant within one band (Band 11, Figure 7C). Pitch oscillation caused a significant increase in the power of the bands encompassing the stimulus frequency (Band 11) and twice the stimulus frequency (Band 10) in BP, although this increase was smaller than that during sGVS (cf BP in Figures 7A,C). In HR, there was similar activation by pitch and sGVS, but there was less power in the band containing twice the stimulus frequency (Band 10, cf HR in Figures 7A,C). There was also activity in Bands 7–9, but it was similar to that observed with no vestibular stimulation (Figure 6D). When animals were pitched at a frequency of 0.1 Hz (Figure 7D), the stimulus frequency was limited to a single band (Band 9). The maximal power of BP and HR were in that band, and the activity in all other bands was smaller and comparable to that with the animal at rest (Figure 6D). Consequently, the wavelet analyses demonstrated that the major oscillations in BP and HR that were associated with frequencies that activated vasovagal responses resided in Bands 10 and 11. This was true regardless of whether vasovagal responses were induced by pitch or by monaural or binaural sGVS. Therefore, they are in the frequency range from 0.018 to 0.035 Hz, which encompasses 0.025 Hz, the best frequency for induction of vasovagal responses.

## Discussion

This study demonstrates that the co-modulation of BP and HR at low frequencies is an essential component of the process that culminates in a vasovagal response. Using sGVS, the optimal frequencies for generation of vasovagal oscillations and for induction of vasovagal responses were 0.025–0.05 Hz. Although the oscillations in pitch did not induce vasovagal responses, the same stimulus frequency range was maximally effective in inducing vasovagal oscillations. In the optimal stimulus frequency range, both BP and HR oscillated at the frequency of stimulation and at twice the frequency of stimulation (double oscillations). The double oscillations disappeared and the single oscillations were attenuated at higher stimulus frequencies.

The results are based on the use of anesthetized rats as a model for human vasovagal responses. There is substantial evidence that the baroreflex is inactivated by isoflurane anesthesia (23), but the sympathetic system overall is unaffected (29). Under normal conditions, the baroreflex reduces the activity of the VSR in order to maintain stable BP. Under anesthesia, with the attendant reduction in sensitivity of the baroreflex, it was possible to elicit the full expression of the vestibulo-sympathetic responses. We posit that the reduction in baroreflex sensitivity is a critical step in allowing the VSR to have expanded access to the cardiovascular system. For example, single sinusoids of sGVS produced strong, concurrent rises in BP and HR that slowly dissipated over several minutes (Figure 3). This is the expected response to elevation of the head and body in a gravitational environment, which produces increases in BP and HR to maintain orthostasis. However, the change in BP induced by the VSR is smaller when the baroreflex is intact (30). It is likely that this reduction in baroreflex sensitivity allowed the emergence of the vasovagal oscillations and vasovagal responses. Exactly how this baroreflex inactivation occurs, however, is unknown.

The most effective frequency to induce vasovagal oscillations and vasovagal responses using sGVS was 0.025 Hz (1 cycle/40 s). Vasovagal oscillations became attenuated, and double oscillations and vasovagal responses disappeared at higher frequencies of sGVS and pitch oscillation. Consequently, 0.025–0.05 Hz was the optimal frequency range for inducing vasovagal responses in the anesthetized rats, consistent with our previous studies (7, 8). Similar low frequency oscillations in BP are found in alert and anesthetized cats and dogs under normal conditions (31), after withdrawal of blood (20), following reductions in arterial BP, and/or by blockade of the renin angiotensin system (32–34). From this, we postulate that there is a specific band of frequencies associated with vasovagal oscillations that have the highest probability of inducing a vasovagal response and vasovagal syncope.

Significantly, vasovagal oscillations could occur without the generation of a vasovagal response, but vasovagal responses were always associated with a vasovagal oscillation. This finding provides strong support for the hypothesis that oscillations in BP are an essential component of the process that generates vasovagal responses and syncope (22). Our data show that the process is more complex because it involves the co-modulation of both BP and HR. Thus, the vestibular system acting through the vasovagal response is capable of provoking the cardiovascular system into low frequency oscillations that can result in vasovagal responses and syncope.

Taken together, we propose that the vasovagal oscillations at low frequencies that are associated with vasovagal responses comprise a unique set of frequencies that constitute a “distress reaction,” which occurs in response to severe anxiety, blood loss, and/or pain. This intense activation of the sympathetic system is manifest in low frequency oscillations of the cardiovascular system and fainting. Under normal circumstances, feedback from the baroreflex contributes to oscillations in BP over a wide range of frequencies (0.02–0.4 Hz). The strongest correlation between BP and HR is at the mid- and high-frequency ranges, however, suggesting that oscillations at frequencies below 0.1 Hz are largely under sympathetic control (31). Why linear acceleration acting through the vestibular/otolith system should have such a powerful influence on generating vasovagal responses and syncope (8) is an obvious but unanswered question. One possibility is that the alterations in BP and HR that lead to syncope cause restoration of normal cardiovascular function. Regardless, the capability of the vestibular system to elicit syncope and pre-syncopal conditions has proven particularly valuable as a diagnostic test (35, 36).

A discrete wavelet analysis was used to analyze the BP and HR signals (22, 37). A wavelet transform computes the similarity between a scaled mother wavelet function with the original signal, and produces coefficients that can be analyzed in the frequency and time domains. This permitted determination of the various frequencies embedded in BP and HR, how they corresponded to the frequency of stimulation, and how these frequencies changed over time. An advantage of wavelet analysis is that the size of the analysis window is determined automatically to give optimal resolution of both time and frequency.

The discrete wavelet decomposition based on Daubechies (Db) wavelets decomposed the original signal into a series of frequency bands that could be examined independently in the time domain. The benefit of the discrete wavelet decomposition was that it produced a finite number of bands and there was no redundancy in the decomposition. Moreover, the original signal could be reconstructed by summing the various bands. Then, the strength of each frequency band was represented by the power of the signal component. The frequency bands were constructed so that they consecutively decreased by a factor of 2, i.e., each higher level band represented the frequency of one half of the previous level. With appropriate resampling, the stimulus was limited to one frequency band, and the power distribution of the BP and HR responses could be determined without bleeding of power across bands.

Monaural and binaural sGVS induce similar oscillations in BP and HR, demonstrating that cathodal activation of one labyrinth can induce both single and double oscillations, without the participation of the other ear. The similarity between the responses to sGVS and oscillation in pitch show that double oscillations are due to otolith processing and not the cathodal activation (24, 25). sGVS pre-dominantly activates the otolith system, although there can also be activation of the vertical semi-circular canals and body tilt receptors (38–41). Prominent features of this otolith activation include the low frequency, double oscillations in response to single sinusoids (e.g., Figure 3), as well as the single oscillations that were revealed by the wavelet analysis and are apparent at higher frequencies of stimulation (Figures 2B and 4C). These results raise the question of how the otolith system produces single and double oscillations of BP and HR.

Oscillations in pitch provide a clear source of data addressing this question. Some primary otolith afferents have polarization vectors close to the vertical axis of the head (42, 43). During pitch oscillation, these neurons are activated twice during each cycle of oscillation as their orientation vectors pass through the spatial vertical. This produces low frequency double oscillations that are conveyed to otolith-recipient central vestibular neurons. We postulate that such afferents can provide the second harmonic that accompanies oscillations in pitch. This would not explain the double oscillations that occur during sGVS, since the entire vestibular nerve is activated by galvanic vestibular stimulation (44). However, the lateral semi-circular canal-recipient central vestibular neurons are inhibited shortly after activation by feedback inhibition (45). The remaining neurons, presumably including those from the otolith system, continue to fire in response to the galvanic stimulation. Among such central otolith-related neurons are those with orientation vectors close to the vertical axis of the head (46, 47). Such neurons would have firing rates that have two peaks during a single cycle of natural stimulation, as their orientation vectors pass through the spatial vertical twice during each cycle. These neurons also have low frequency characteristics, so they could be responsible for the low frequency properties of the double oscillations induced by frequencies close to 0.025–0.05 Hz. Other central otolith neurons, with different orientation and frequency characteristics, could produce the single response frequencies as the activation frequencies approach 0.1 Hz. Such “single frequency” neurons may play an important role in the generation of vasovagal responses as shown in Figure 8. We speculate, therefore, that a combination of the activity from several classes of peripheral and central otolith system neurons provides the activation for the VSR that produces both the increases in BP and HR when the system is functioning normally and the activation of vasovagal oscillations when the baroreflex is inactivated.

The question remains, do these central otolith-related vestibular neurons convey signals to pre-sympathetic neuronal pools that control changes in BP and HR, and if so, do they have similar functional characteristics? Such connections were demonstrated using cFos protein to identify vestibular neurons activated by sGVS (26). The sGVS-activated cells were concentrated in the caudal inferior and medial vestibular nuclei, otolith-recipient regions, and sent axonal projections to the rostral and caudal ventrolateral medullary areas (48). These regions are integral parts of the sympathetic pathway to the spinal cord, ultimately leading to activation of the blood vessels and the heart [see Ref. (49) for review]. Of interest, the majority of neurons in the rostral ventrolateral medulla that receive vestibular input are otolith and not semi-circular canal-related (50). Although polarization vectors of rostral ventrolateral medullary neurons are equally distributed in various vertical planes from pitch to roll (40), the optimal vestibular modulation of sympathetic spinal neurons is in the pitch plane (41, 51). Thus, there appears to be coherence of the neuronal types from the otolith organs to the effector cells in the spinal cord that actually produce the changes in BP and HR, including vasovagal oscillations.

### Summary

Based on findings in the anesthetized rat, we propose that there is a specific low frequency band that contains activity that triggers the cardiovascular system into oscillation, and that these oscillations are critical for the generation of vasovagal responses in rats, and presumably vasovagal responses and syncope in humans. We further hypothesize that the otolith system is the major pathway from the vestibular to the autonomic system that is responsible for vestibularly induced neurogenic syncope.

## Conflict of Interest Statement

The authors declare that the research was conducted in the absence of any commercial or financial relationships that could be construed as a potential conflict of interest.

## Abbreviations

BP, arterial blood pressure; bpm, beats per minute; double oscillations, dominant second harmonic, i.e., oscillation at twice the stimulus frequency; HR, heart rate; MSNA, muscle sympathetic nerve activity; power distribution, the relative magnitudes of the frequencies comprising an induced waveform; sGVS, sinusoidal galvanic vestibular stimulation; VSR, vestibulo-sympathetic reflex; VVO, vasovagal oscillations (in blood pressure and heart rate); VVR, vasovagal response.
